# Recurrent Aseptic Meningitis Revealing Primitive Pituitary Abscess

**DOI:** 10.7759/cureus.78537

**Published:** 2025-02-05

**Authors:** Hadhami Ben Turkia, Marwa Sameer

**Affiliations:** 1 Pediatrics, King Hamad University Hospital, Muharraq, BHR

**Keywords:** case report, meningitis, paediatrics, pituitary abscess, pituitary disorders

## Abstract

We report a case of primitive pituitary abscess (PA) in a teenage girl who was admitted on three occasions for acute meningitis. A lumbar puncture revealed aseptic meningitis and the brain MRI showed a well-defined intrasellar space-occupying lesion with suprasellar extension suggestive of macroadenoma. The patient remained free of symptoms between episodes of meningitis however she developed a diabetes insipidus. The diagnosis of PA was established preoperatively but no microorganisms were isolated. Postoperatively she developed pan-hypopituitarism. Her growth velocity remained normal. At the age of 17 years, she was started on somatotropin in view of low insulin-like growth factor-1 (IGF1) level.

This case illustrates an insidious presentation of PA in a child without apparent risk factors and complicated with hypopituitarism. Early diagnosis and treatment are crucial to prevent life-threatening complications and irreversible pituitary dysfunction.

## Introduction

Pituitary abscess (PA) is defined by the presence of an infected purulent collection within the sella turcica. PA can be classified as primary if pituitary gland is confirmed normal, or secondary when associated with an underlaying sellar pathology before the infection [1]. It is rare and accounts for 0.2-1.1% among operative pituitary lesions [2]. Agyei et al. compiled 200 published cases in 2017, in which only 23 cases were younger than 18 years [3]. Gao et al. reported the largest series of 66 cases compiled over 23 years in Japan [4]. Mallereau et al. reported a series of 84 primary PAs collected from an European multi-center study over 20 years [1]. The main presenting features of PA are headache, pituitary dysfunction, and visual disturbances [2-4]. Diagnosis is often difficult before surgery due to the lack of specific clinical symptoms and signs, as well as radiologic similarities with other pituitary lesions [3,5]. Herein, we report an atypical and insidious presentation of primary PA in an immunocompetent child.

## Case presentation

A 10-year-old girl presented with a fever, headache and vomiting, which had started one day ago. She was not known to have any medical conditions and was fully immunized. Physical examination revealed high grade fever, meningeal signs and altered mental status with a Glasgow Coma Score of 12 which required her admission in the pediatric ICU.

**Figure 1:**
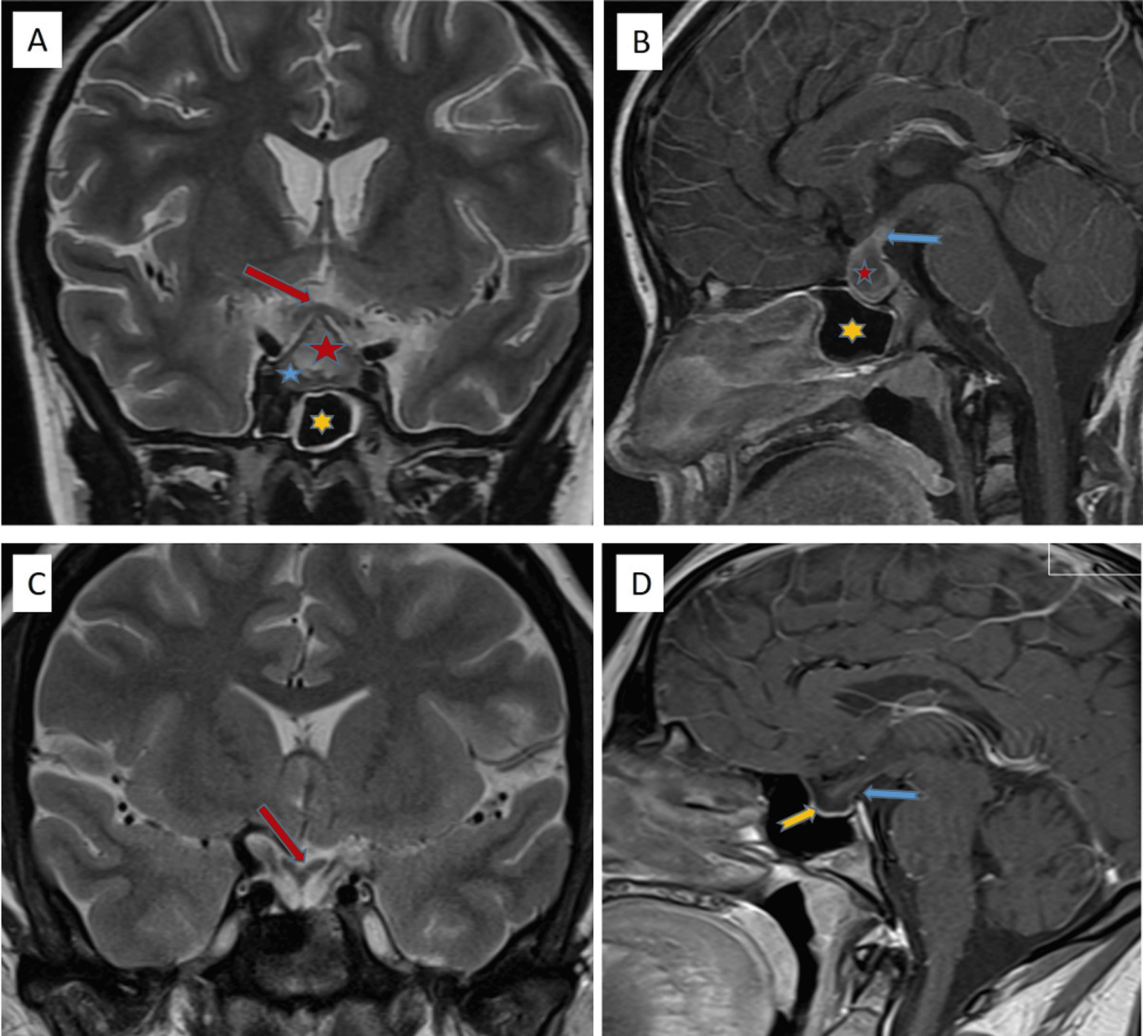
Brain MRI pre and post surgery A,B: Pre-surgery images; C,D: Post-surgery images A: T2 coronal image; inhomogeneous hyperintense, well-defined focal area (red star) in the anterior pituitary gland, and indentation on optic chiasm with cranial displacement (red arrow). PA. Normal glandular tissue is seen as a thinned area at caudal level (blue star). Mucosal thickenings are seen at walls of sphenoid sinus (yellow star). B: Post-contrast T1 sagittal image; thickened stalk (blue arrow) and peripherally irregular contrast enhancement is seen on PA (red star). Sphenoid sinus is represented by the yellow star. C: T2 coronal post-surgery image; caudally displacement/retraction of optic chiasm and stalk (red arrow) and significant volume loss at anterior pituitary gland (yellow arrow). D: Post-contrast T1 sagittal image; significant volume loss of anterior pituitary gland (yellow arrow) and thinned stalk (blue arrow). Empty sella findings. PA: Pituitary abscess

The first brain MRI showed an intrasellar lesion 19 x 16 x 12 mm with peripheral enhancement and suprasellar extension suggestive of a pituitary macroadenoma. There was no abnormal meningeal enhancement in the post-contrast study and no areas of restricted diffusion on diffusion weighted images (Figures 1A, 1B).

The lumbar puncture failed and blood culture showed no growth. She fully recovered after a 10-day course of antibiotics (ceftriaxone and vancomycin) and the initially high C-reactive protein (CRP) (169 mg/l) dropped to 6.5 mg/l. The results of the pituitary function assessment, including adrenocorticotropic hormone (ACTH), cortisol, growth hormone, free thyroxine (FT4), thyroid-stimulating hormone (TSH), luteinizing hormone (LH), follicle-stimulating hormone (FSH), and low-dose Synacthen test, were normal (Table 1).

**Table 1:** Hormonal profile pre and post surgery TSS: Transsphenoidal surgery; PS: Post surgery; ND: Not done; NA; Not applicable; ACTH: Adrenocorticotropic hormone;  FT4: Free thyroxine; TSH: Thyroid-stimulating hormone; IGF1: Insulin-like growth factor-1; FSH: Follicle-stimulating hormone; LH: Luteinizing hormone *First Synacthen Test (Low dose): Baseline: 0.52 mcg/dL, 30 min: 3 mcg/dL, 60 min: 4.6 mcg/dL **Second Synacthen Test (250 mcg):  Baseline: 7 mcg/dL, 30 min: 9.25 mcg/dL, 60 min: 24.9mcg/dL

Presentation		1st Meningitis	2nd Meningitis	3rd Meningitis	TSS	2m PS	7m PS	3.5y PS	5y PS	6y PS	6.5y PS	7y PS	7.5y PS
Age (y)		9.5	9.5	10		10.5	12	14	15	17	17	17.5	18
Hormonal profile pre and post surgery		Pre surgery				Post surgery							
Cortisol (a.m.) (mcg/dl)	6.99-25	Normal	Normal	Low		<0.5	-	8.59	7	11	11	2	2.8
ACTH (pg/ml0	10-48	Normal	Normal	Normal		25	21.3	ND	18	ND	ND	ND	ND
FT4 (ng/dl)	0.47-1.99	Normal	Normal	Normal		0.75	1.35	0.61	0.7	0.75	0.83	0.93	0.69
TSH (UIU/ml)	0.35-5.5	Normal	Normal	Normal		0.089	0.018	4	4.3	4.41	4.2	0.37	4
IGF1 (IU/ml)	226-903	ND	ND	ND		ND	ND	NA	297	22	32.5	20	ND
FSH (mUI/ml)		Normal	ND	ND		ND	ND	NA	ND	ND	ND	0.27	ND
LH (mUI/ml)		Normal	ND	ND		ND	NA	NA	ND	ND	ND	0	ND
Synacthen Test*		ND	Normal	Abnormal*		ND	ND	ND	ND	Normal **	ND	ND	ND
Polyuria/polydipsia		Nil	++	Nil		Nil	Nil	Nil	Nil	Nil	Nil	Nil	Nil
Medications													
Desmopressin (20 mcg OD)			+	+		+	+	+	+	+	+	+	+
Levothyroxine (100 mcg OD)			-	-		-	+	+	+	+	+	+	+
Hydrocortisone (20 mg OD)			-	+		+	+	-	-	-	-	+	+
Estradiol (1 mg OD)			-	-		-	-	+	+	+	+	+	+
Somatotropin (0.3 mg OD)			-	-		-	-	-	-	+	+	-	-

She presented six weeks later with the same symptoms. Following is the result of CSF analysis: pleocytosis (1,300/mm^3^), 50% of which were polymorphs, hypoglycorrhachia (low glucose in CSF, as CSF glucose to serum glucose was low (0.08)) and high protein concentration. Blood and CSF cultures were negative. She received a two-week course of ceftriaxone and vancomycin, the latter was discontinued after she developed red man syndrome. After recovery, she developed diabetes insipidus but the rest of the pituitary hormones were tested normal. 

The third meningitis occurred eight months later and repeated brain MRI showed a well-defined intrasellar cyst-like lesion of 15 x 20 x 16 mm with ring enhancement and suprasellar extension, obliteration of the suprasellar cistern and the cavernous sinuses with a mild compression of the optic chiasm and a thickened pituitary stalk. Minimal mucosal thickening of the left compartment of the sphenoid sinus was noted. She received a two-week course of linezolid resulting in full clinical recovery. The child was asymptomatic between the meningitic episodes and did not complain of any headaches or visual disturbances.

In view of recurrent meningitis; serum immunoglobulins, complement fractions, lymphocytes subsets and technetium 99m-diethylenetriamine penta-acetic acid (DTPA) brain for CSF leak were performed and came out to be normal. Low-dose ACTH test showed a suboptimal cortisol response and the patient was started on hydrocortisone replacement. The patient underwent transsphenoidal exploration that revealed a thick-walled abscess containing yellowish pus, which was drained and excised. Histology showed fibrohyalinized and collagenized tissue with diffuse mixed inflammatory cell infiltration composed of polymorphs and lymphocytes. Nests of larger cells were observed within some of the bits representing the anterior pituitary gland and pars intermedia. The synaptophysin strain was strongly positive for the pituitary cells. The culture and Ziehl-Neelsen test results were negative. Postoperatively, intravenous antibiotic (linezolid) was continued for a total of one month.

After surgery, she developed thyreotrope and gonadotrope deficiencies. Estrogen therapy was initiated at the age of 14 years with regular menstruation. Corticotropic deficiency resolved within eight months after surgery and the cortisol levels remained normal up to six years after surgery with a normal response to standard ACTH test (Table 1). The patient was restarted on hydrocortisone seven years post surgery in view of low cortisol levels. Her growth velocity remained normal (height on the fiftieth percentile). Insulin-like growth factor-1 (IGF1) level, which was initially normal, was found to be low at the age of 17 years. Hence, she was started on growth hormone replacement therapy. The latest brain MRI, done seven years post surgery showed no residual lesion, a pituitary gland height of 1 mm, and a normal stalk thickness suggestive of empty sella (Figures 1C, 1D).

## Discussion

PA is a rare condition in children. In a systematic review by Agyei et al., 23 patients were younger than 18 years, with an average age of 15 years [3]. To our knowledge, this is the youngest reported case. PA may develop from pre-existing pituitary lesion (secondary PA) such as pituitary adenoma, craniopharyngioma, or Rathke’s cleft cyst [4-7]. Such lesions were reported in 30% of cases in Gao et al.'s series [4]. Among the cases compiled by Zegarra-Linares et al., only three of the 11 patients younger than 21 years had an underlying pituitary gland condition [8].

Primary PAs occur in normal pituitary gland resulting from hematogenous seeding, direct extension from an adjacent infectious process, such as sphenoid sinusitis, meningitis, cavernous sinus thrombophlebitis, or after transsphenoidal surgery (TSS) [9,10]. Our patient did not have any of these risk factors. The minimal sphenoidal mucosal thickening noted on MRI was unlikely to be the cause, as the patient denied any symptoms of sinusitis [11]. No obvious reason can be found in 60% of patients [11].

Most commonly, PA has an indolent presentation; however, a rapidly progressive clinical picture has been reported. Fever is not constant and was described in 25% of pediatric cases [8]. Our patient presented with an acute onset of headache, vomiting and impaired level of consciousness that might suggest an apoplexy of the pituitary adenoma, which is rare in children. Furthermore, there was no associated ophthalmoplegia, and MRI findings did not support this diagnosis [12,13]. Additionally, meningitis is an uncommon complication of untreated macroadenoma and is exceptional if there is no CSF leakage through disruption of the sphenoid floor, which was excluded in our patient. A possible explanation for the recurrent meningitis could be a leakage of pus from the pituitary lesion into the subarachnoid space [14]. Our patient did not experience any chronic headache or visual disturbances, although these two symptoms are the most frequent presenting symptoms found in pediatric cases with frequencies of 78% and 43%, respectively [3].

In the systematic review by Agyei et al., 82% of patients presented with endocrine abnormalities, and only four from the pediatric group had normal hormonal profiles on presentation [3]. After treatment, the pituitary dysfunction may not normalize; for instance, approximately 50% of pediatric cases with preoperative pituitary dysfunction had partial or no endocrine recovery in the systematic review. Higher recovery of pituitary hormone function is observed in patients with a short duration of symptoms and those with primary abscesses as opposed to secondary ones [3,5,6,8,15]. The preoperative corticotropic deficiency documented in our patient with low-dose ACTH test resolved within eight months after surgery and the patient remained asymptomatic for years. Furthermore, standard ACTH test performed six years post surgery came to be normal until cortisol levels dropped one year later. Indeed, secondary corticotropic deficiency is usually less severe than the primary and the standard ACTH test might resulted in supra-physiologic adrenal stimulation and led to false positive test. Studies concluded that low-dose ACTH of 0.5 to 1 mcg test should be preferred in suspected secondary adrenal insufficiency as it has better sensitivity than the standard-dose [11].

Trans-sphenoidal drainage is the treatment of choice, and antibiotic therapy, directed by culture, should be administered for four-six weeks. Among the 84 patients reported in the multi-center study, a bacterial agent was identified in only 25% that might be related to empiric preoperative antibiotic therapy. *Staphylococcus aureus* and Streptococcus species are the most frequent bacteria isolated [1,3]. In our patient, both CSF and drained pus cultures were sterile. However, histopathology examination demonstrated an inflammatory infiltration without evidence of other pituitary pathologies.

## Conclusions

The clinical presentation of our case was atypical, with isolated recurrent aseptic meningitis and a symptom-free interval of over a year delaying the diagnosis and compromising the pituitary function. PA should be considered for any cystic intrasellar lesion, even in the absence of infective signs. Early diagnosis and treatment are crucial to prevent life-threatening complications, such as neurological deficits, sepsis, or even death.
